# Temporal Trends and Risk Factors for Delayed Hospital Admission in Suspected Stroke Patients

**DOI:** 10.3390/jcm9082376

**Published:** 2020-07-25

**Authors:** Moritz Kielkopf, Thomas Meinel, Johannes Kaesmacher, Urs Fischer, Marcel Arnold, Mirjam Heldner, David Seiffge, Pasquale Mordasini, Tomas Dobrocky, Eike Piechowiak, Jan Gralla, Simon Jung

**Affiliations:** 1 Department of Neurology, Inselspital, Bern University Hospital, University of Bern, 3010 Bern, Switzerland; moritz.kielkopf@insel.ch (M.K.); thomas.meinel@insel.ch (T.M.); urs.fischer@insel.ch (U.F.); marcel.arnold@insel.ch (M.A.); mirjam.heldner@insel.ch (M.H.); david.seiffge@insel.ch (D.S.); 2 Institute of Diagnostic and Interventional Neuroradiology, Institute of Diagnostic, Interventional and Pediatric, Radiology and Department of Neurology, University Hospital Bern, Inselspital, University of Bern, 3010 Bern, Switzerland; johannes.kaesmacher@insel.ch; 3 University Institute of Diagnostic and Interventional Neuroradiology, Inselspital, Bern University Hospital, University of Bern, 3010 Bern, Switzerland; Pasquale.Mordasini@insel.ch (P.M.); Tomas.dobrocky@insel.ch (T.D.); eike.piechowiak@insel.ch (E.P.); Jan.gralla@insel.ch (J.G.)

**Keywords:** time to admission, prehospital delay, stroke, prior stroke

## Abstract

(1) Background: The benefit of acute ischemic stroke (AIS) treatment declines with any time delay until treatment. Hence, factors influencing the time from symptom onset to admission (TTA) are of utmost importance. This study aimed to assess temporal trends and risk factors for delays in TTA. (2) Methods: We included 1244 consecutive patients from 2015 to 2018 with suspected stroke presenting within 24 h after symptom onset registered in our prospective, pre-specified hospital database. Temporal trends were assessed by comparing with a cohort of a previous study in 2006. Factors associated with TTA were assessed by univariable and multivariable regression analysis. (3) Results: In 1244 patients (median [IQR] age 73 [60–82] years; 44% women), the median TTA was 96 min (IQR 66–164). The prehospital time delay reduced by 27% in the last 12 years and the rate of patients referred by Emergency medical services (EMS) increased from 17% to 51% and the TTA for admissions by General Practitioner (GP) declined from 244 to 207 min. Factors associated with a delay in TTA were stroke severity (beta−1.9; 95% CI–3.6 to −0.2 min per point NIHSS score), referral by General Practitioner (GP, beta +140 min, 95% CI 100–179), self-admission (+92 min, 95% CI 57–128) as compared to admission by emergency medical services (EMS) and symptom onset during nighttime (+57 min, 95% CI 30–85). Conclusions: Although TTA improved markedly since 2006, our data indicates that continuous efforts are mandatory to raise public awareness on the importance of fast hospital referral in patients with suspected stroke by directly informing EMS, avoiding contact of a GP, and maintaining high effort for fast transportation also in patients with milder symptoms.

## 1. Introduction

The benefit of acute ischemic stroke (AIS) management is strongly time dependent. Using advanced imaging techniques, the time window for recanalization therapies has extended over the last decades. Thrombolysis has shown to improve outcome in selected patients up to 9 h after symptom onset [1], while endovascular thrombectomy lowered disability in selected patients with a large vessel occlusion up to 16–24 h after onset [2,3]. However, for endovascular treatment and thrombolysis [4,5,6] as well as conservative medical management [7] the time from symptom onset to initiation of therapy remains a decisive factor for functional outcome [6]. While efforts to reduce the door-to-treatment time have led to significant improvements [8], the development of prehospital delays in AIS remains controversial. Various global reviews reported divergent results with regard to the prehospital time improvement and the factors leading to delay [9,10,11]. For example, the impact of a previous cerebrovascular event (pCVE), i.e., previous stroke or transient ischemic attack (TIA) in the patient medical history on the time to hospital admission (TTA) remains uncertain. Additionally, there is a lack of data on the impact of optimized prehospital workflows.

Therefore, the aim of the study was to assess factors associated with TTA delays of suspected stroke patients referred to the Stroke Center Bern. In addition, we aimed to analyze temporal trends of TTA delay in comparison with a previous study cohort in 2006 [12].

## 2. Experimental Section

### 2.1. Material and Methods 

**Figure 1:**
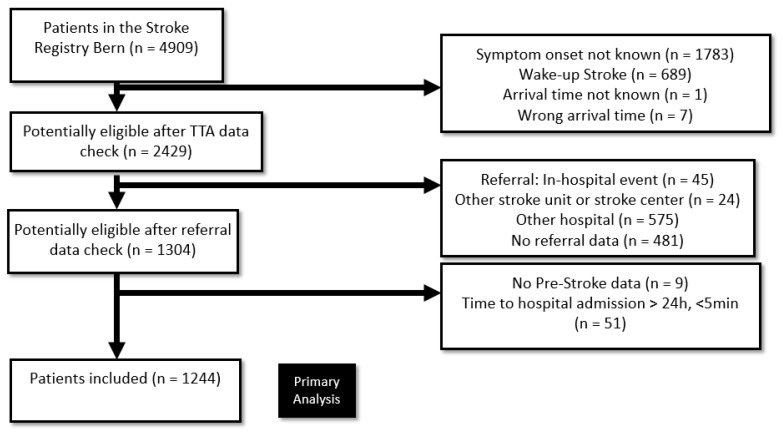
Inclusion Criteria in the main analysis.

Consecutive patients with a final diagnosis of AIS, TIA, amaurosis fugax, cerebral venous thrombosis or stroke mimics were analyzed. These events may typically present with symptoms compatible with AIS and demand the same diagnostic procedure as well as the ignition of the so called, stroke chain of survival [8]. Patients treated at emergency department from 01 February 2015 to 26 December 2018 were included. Demographic data and baseline variables were collected prospectively in our Bernese Stroke Database. The primary outcome variable (TTA) was defined as the time from onset of neurological symptoms to the time of hospital arrival. In the database, hospital arrival is declared as the time when patients were registered at the triage of the Emergency Department. We included patients presenting between 5 min and 24 h after symptom onset. Exclusion criteria were in-hospital strokes, wake-up strokes, as well as inter-hospital referrals. All inclusion and exclusion criteria and inclusion chart are presented in Figure 1.

To asses temporal trends in TTA delay we compared our data with a previous study in 2006. To ensure comparability we applied the same inclusion criteria to our dataset as in 2006 in a sub analysis. Therefore we included inter-hospital referrals (n = 652) and wake up strokes in the sub analysis. In patients with wake up stroke and patients who were found unconscious or aphasic, the wake up time or time of finding the patients was considered as the start of TTA. Furthermore, in subgroup analysis, the time span was restricted to a maximum of 48 h after symptom onset and only ischemic strokes and TIAs were included. 

### 2.2. Statistical Analysis 

To assess which factors were associated with prehospital delays, we compared patients with a fast (<96 min) and a long (>96 min) TTA using the median TTA as the cut-off point. To determine significant differences, we used appropriate statistical measures (χ^2^ test for categorical variables, Fisher’s exact test for categorical variables, Mann-Whitney-U-Test for non-normally continuous or ordinally scaled variables, and Welch’s t-test for independent normally distributed data). To document the variance of continuous variables, we present results as median and interquartile range (IQR). We included variables with a *p*-value of <0.2 (statistical criterion), analyzed (multi)collinearity between variables and hence selected pathophysiologically plausible variables for the final multivariate model.

For the primary analysis the association of factors with TTA was assessed using linear regression adjusting for the following confounders: sex (categorical), age (continuous), diabetes mellitus (categorical), referral by GP as compared to EMS (categorical), self-referral GP as compared to EMS (categorical), presentation during daytime (categorical), stroke severity (NIHSS on admission [13], ordinal), systolic blood pressure on admission (mmHG, ordinal) and pCVE (categorical). We calculated (adjusted) beta regression coefficients (β) and corresponding 95% confidence intervals. All statistical analyses were performed using SPSS (IBM Corp. Released 2017. IBM SPSS Statistics for Windows, Version 25.0. Armonk, NY, USA: IBM Corp.). All *p* values are 2-sided, with *p* < 0.05 considered statistically significant. No adjustments for multiple testing were applied and patients with missing data items were excluded from the multivariate analysis.

## 3. Results

**Figure 2:**
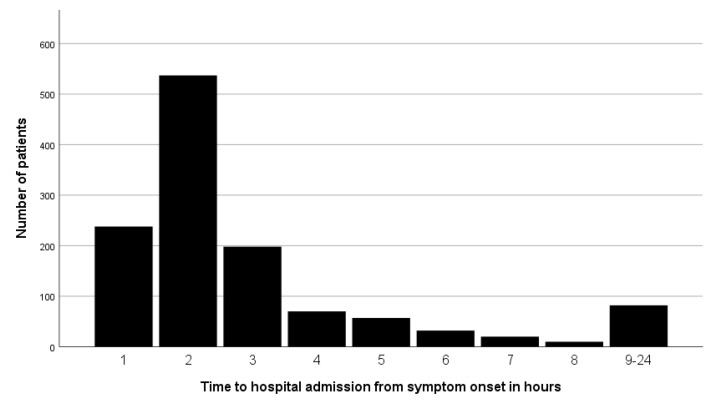
Prehospital delay time of patients with acute cerebrovascular event in hours.

Of 4909 patients in the registry, 1244 patients complied with the inclusion criteria and were included in this analysis (see Figure 1 for reasons of exclusion). Median age was 73 years (60–82), 44% were female, median NIHSS score 4 (1–11). In total, 62.6% of patients presented within 0–2 h after symptom onset, 21.3% of patients within 2–4 h, 7.1% within 4–6 h, 2.4% within 6–8 h and only 6.5% of patients beyond 8 h after symptom onset (Figure 2). 81% of patients had an AIS as final diagnosis, whereas 19% suffered from other vascular emergencies (TIA, amaurosis fugax, cerebral venous thrombosis or stroke mimics). The median TTA was 96 min (IQR 66–164).

Of 4909 patients in the registry, 2088 patients complied with the inclusion criteria from 2006 as mentioned above. The median TTA was 132 min (IQR 79–244) in comparison to 180 min in 2006 and 86% patients arrived within 6 h (75% in 2006). In 2018 51% of this patient group were referred by EMS compared to 17% in 2006. The exact comparison to the study conducted in 2006 is shown in Table 1.

### Factors Associated with TTA Delay

In univariable analysis the following factors were significantly associated with shorter TTA: referral by EMS, ischemic stroke event type, more severe stroke according to the NIHSS score, and higher systolic blood pressure on admission. Additionally the number of patients with a medical history of diabetes or with at least three vascular risk factors was significantly lower in the fast arrival group. Baseline characteristics of patients according to time from symptom onset to hospital admission are shown in the Appendix A, Table A1.

Variables associated with TTA in multivariable analysis were stroke severity (beta −1.9; 95% CI −3.6 to −0.2 min per point NIHSS score), referral by General Practitioner (GP) as compared to admission by EMS (beta + 140 min, 95% CI 100–179), self-admission as compared to admission by EMS (+92 min, 95% CI 57–128) and symptom onset during nighttime (+57 min, 95% CI−85 to−30, Table 2). There was no significant association of age, sex, blood pressure, and diabetes in the multivariable linear regression model. (Table 2).

In total, 319 patients (26%) were not referred by EMS of whom 134 were referred by GP (11%) and 185 by self-referral (15%). Patients not primarily admitted by EMS had less severe stroke (median NIHSS score 1, IQR 0–3 vs. 5, 2–11, *p* < 0.001), and were younger (67 years, 50–78 vs. 74, 62–83, *p* < 0.001). 302/1244 (24.3%) of the included patients have already suffered a pCVE. As compared to patients without pCVE, these patients were older, had a different referral pattern, more often a pre-existing disability, a worse vascular risk profile, and more often preceding antithrombotic therapy.

There was no difference in TTA between patients with a pCVE (94 min, IQR 64–160) as compared to patients without a pCVE (100 min, IQR 70–183, *p* = 0.078) in univariate analysis. After adjustments for confounders, pCVE was not associated with TTA (beta +1 min, 95% CI −26–+29 min).

## 4. Discussion

The main findings of our registry-based study of 1244 patients with acute vascular events presenting between 2015 and 2018 are:

(1) The median TTA was 96 min (IQR 66–164). (2) The prehospital time delay reduced by 27% in the last 14 years. (3) Self-referral or referral by a GP, lower NIHSS score and nighttime symptom onset were associated with a delay in TTA in both univariable and multivariable analysis. (4) Referral by EMS was the only modifiable variable associated with shorter TTA in both univariable and multivariable analysis. (5) A pCVE had no influence on TTA neither in univariable nor multivariable analysis.

Our results demonstrate that prehospital delay in patients with symptoms compatible with an acute vascular emergency are still considerable. The median TTA was around 1.5 h in our study, which is among the shortest reported prehospital times in different stroke networks, but is still too long [9,14]: Whereas 63% of patients arrived within 2 h, one of five patients (22%) arrived later than 3 h after symptom onset.

Previous studies on temporal trends of TTA revealed conflicting results. Whereas some studies indicated an improvement in TTA over the years [10,15], a meta-analysis on global cross-sectional studies could not find an improvement in TTA [9]. In this context the adj. TTA analyzation was carried out to carefully investigate time trends based on the same geographic, infrastructural and social characteristics. In the adjusted subgroup analysis, the median TTA was 132 min, corresponding to an absolute reduction of 48 min (27%) compared to 14 years ago. The median TTA for patients referred by GP dropped from 224 min in 2006 to 207 min in 2018 and for self-referral patients from 174 min in 2006 to 140 min in 2018 [12]. The fastened referral times are probably at least the the result of the efforts made in stroke information campaigns in the Bernese region. The key messages of the information campaigns were to avoid GP contact when stroke symptoms occur and to refer severe affected patients direct to a Stroke-Unit center. Indeed, the referral rate by EMS increased from 17% in 2006 to 51% in 2018 whereas GP referrals decreased from 38% to 7% and inter-hospital referrals from 38% to 31%.

The EMS referral times itself did not change over time. This might indicate that this referral pathway might already be close to its optimal speed. Our data indicates that continuous efforts are mandatory to raise public awareness on the importance of fast hospital referral in patients with suspected stroke by directly informing EMS, avoiding presentation at a GP, and maintaining high effort for fast transportation also in patients with milder symptoms.

The analysis of factors that contribute to TTA delays revealed referral by GP, self-referral, nighttime event and low NIHSS score. Referral by GP was associated with an approximated time delay of 2 h, self-referral with a time delay of 1.5 h and wake up stroke with 1 h delay. Our findings are in line with multiple studies investigating the impact of EMS over the last 10 years [16,17,18]. Age, sex and vascular risk factors had no significant influence in our multivariate analysis.

In concordance with the results from 2006, patients with pCVE showed a non-significant trend to longer TTA. At first glance, this seems surprising as these patients presumably have been informed about cerebrovascular diseases and the time is brain concept. Nevertheless, this is in line with previous studies suggesting that better stroke knowledge does not necessarily increase EMS use [14,16,19,20].

### Limitations and Strengths

The strengths of our study are the size of the cohort, and the longitudinal comparison within the same network/population after several years. A limitation is a potential bias due to the exclusion of patients with unknown symptom onset, who potentially tend to show a TTA above average (n = 1783). In addition, the overall GP referrals in 2006 included 64/233 patients from so called Emergency doctors (ED), a system of familiar physicians who take regular terms in an emergency service outside the hospital, which restricts in some way the comparability of the two cohorts.

## 5. Conclusions

Referral by EMS, high NIHSS scores and symptom onset during daytime are independently associated with shorter TTA. The median TTA was 96 min, resulting in considerable improvement compared to our previous study roughly 10 years ago, mainly due to an increase of the referrals by EMS (17% to 51%). Continuous efforts are mandatory instructing stroke patients with vascular risk factors and their relatives, as well as GPs, to immediately request EMS assistance in case of suspected stroke.

## Figures and Tables

**Table 1:** Comparison of adj. TTA and adj. referral pathways in acute stroke 2018 with 2006.

	2018	2006
EMS referral	51% (1063/2088)	17% (105/615)
GP referral *	7% (151/2088)	38% (233/615)
Self-referral	11% (222/2088	12% (46/615)
Inter-hospital referral	31% (651/2088)	38% (231/615)
Median TTA	132 min	180 min
EMS referral Ambulance (Median TTA) Helicopter (Median TTA)	88 min 99 min	80 min 93 min
GP ** (Median TTA)	207 min	224 min
SR (Median TTA)	140 min	174 min
Inter-hospital (Median TTA)	209 Min	195 min

The data from 2006 are the results of a previous study analyzing the time to admission in AIS, conducted within the same stroke network. * The data of 2006 include 64/233 patients referred by so-called Emergency doctors (ED), a system of familiar physicians who take regular terms in an emergency service outside the hospital. ** In 2006 only patients with direct GP-referral (n = 169) are considered for exact delay comparison (ED referrals excluded). SR, self-referral; GP, general practitioner; EMS, emergency medical services; TTA, time to hospital admission.

**Table 2:** Factors associated with time to admission (linear regression analysis).

Variable	Beta	95% CI	*p*-Value
Sex	16.5	−7.0–40.0	0.169
Age	−0.2	−1.0–0.6	0.589
Diabetes Mellitus	12.5	−19.4–44.5	0.442
General Practitioner	139.7	100.3–179.2	0.000 †
Self-Referral	92.1	56.6–127.5	0.000 †
Daytime	−57.5	−85.0–(−29.9)	0.000 †
PCVE	1.4	−26.0–28.7	0.921
NIHSS score to admission	−1.9	−3.6–(−0.2)	0.028 †
Blood pressure systolic (mmHg)	0.17	−0.3–0.6	0.434

† Statistically significant. Beta, Regression coefficient in its influence in mins. on TTA; Daytime, Daytime from 7 a.m.–7 p.m. ; PCVE, Previous cerebrovascular event ; NIHSS score, National Institute of Health Stroke Scale.

## Appendix A

**Table A1:** Baseline characteristics of patients according to time from symptom onset to hospital admission.

	n	Hospital Arrival within 96 min	Hospital Arrival after 96 min	*p*-Value
Age (IQR/median)	1244	62.27–82/73.45	57.58–82.1/73.15	0.113
Men (number, %)	1244	352/622 (56.6%)	351/622 (56.4%)	0.954
Referral (number, %) Self Referral Emergency service (144) General Practitioner	1244	73/622 (11.7%) 521/622 (83.8%) 28/622 (4.5%)	112/622 (18%) 404/622 (65%) 106/622 (17%)	0.000 †
Type of event (number, %) Ischemic Stroke Transient ischemic attack Others	1244	524/622 (84.2%) 89/622 (14.3%) 9/622 (1.4%)	483/622 (70.4%) 104/622 (16.7%) 35/622 (5.6%)	0.002 †
Daytime (number, %)	1244	488/622 (78.5%)	463/622 (74.4%)	0.109
PS-Disability mRS (number, %) 0 1 2 3 4	946	324/490 (66.1%) 96/490 (19.6%) 28/490 (5.7%) 31/490 (6.3%) 11/490 (2.2%)	300/456 (65.6%) 87/456 (19%) 30/456 (6.6%) 31/456 (6.8%) 9/456 (2%)	0.975
Medical History (number, %) PCVE Diabetes Hyperlipidemia Hypertension Atrial fibrillationAny of this conditions >3 RF		141/622 (22.7%) 82/621 (13.2%) 396/619 (64%) 435/621 (70%) 191/620 (30.8%) 547/618 (88.5%) 202/618 (32.7%)	161/621 (25.9%) 115/621 (18.5%) 408/616 (66.2%) 429/620 (69.2%) 191/620 (30.8%) 547/610 (89.7%) 236/610 (38.7%)	0.181 0.010 † 0.405 0.743 1.0 0.514 0.028 †
Living situation–at home (number, %)	1240	589/620 (95%)	581/620 (93.7%)	0.325
NIHSS (IQR/median)	1186	2–13/5, n = 609	1–8/3, n = 577	0.000 †
Blood pressure systolic (mmHg) (IQR/median)	1231	140–177/160, n = 615	135–175/155, n = 616	0.032

Others, retinal infarct, amaurosis fugax, sinus vein thrombosis and stroke mimics; mRS, modified Rankin Scale; PS, Pre stroke; PCVE, Previous cerebrovascular event; RF, risk factor; NIHSS score, National Institute of Health Stroke Scale; Daytime, Daytime from 7 a.m.–7 p.m.
